# Meteorological Diary, Kept at Clapton, Hackney, from Feb. 26, to March 27, 1810

**Published:** 1810-05

**Authors:** Thomas Foster


### *Meteorological Diary, kept at Clapton, Hackney, from Feb. 26, to March 27,* 1810, *by* THOMAS FOSTER, Esq.

| Day of M. | | Thermom. Max. | Thermom. Min. | Barometer. Max. | Barometer. Min. | Wind. | Weather. |
|---|---|---|---|---|---|---|---|
| Feb. ☾ | 26 | 48° | 45° | 29 90 | 29 77 | W. | Sun & clouds. Cloudy night |
| | 27 | 54 | 37 | 30 07 | 29 72 | S. W.—W. | Misty & sm. rain, *am.* Fine *p m* |
| | 28 | 54 | 47 | 30 07 | 29 90 | W. | Sun and clouds |
| March | 1 | 55 | 48 | 29 83 | 29 72 | W. S. W. | Misty *a. m.* Sun & clouds *p. m.* |
| | 2 | 54 | 45 | 29 73 | 29 65 | S. W. | Overcast & misty. Rainy nig. |
| | 3 | 53 | 33 | 29 70 | 29 57 | W. | Foggy—rain—clear |
| | 4 | 49 | 40 | 29 59 | 29 30 | S. | Fog—sun & clouds—rain |
| 1 ● 23′ | 5 | 46 | 32 | 29 30 | 28 95 | N. | Cloudy—sun & clouds |
| | 6 | 39 | 35 | 29 | 28 94 | N. W. S. E. | Snow, *a. m.* Clouds—clear |
| | 7 | 45 | 38 | 28 99 | 28 85 | S. E. | Rain, *a. m.* Clouds, *p. m.* |
| | 8 | 51 | 45 | 29 17 | 29 07 | S. | Sun & showers. Clear night |
| | 9 | 57 | 50 | 29 33 | 29 17 | S. W. | Sun & showers. Windy night |
| | 10 | 58 | 46 | 29 89 | 29 15 | W. | Sun & clouds. Clear, *p. m.* |
| | 11 | 57 | 50 | 29 95 | 29 70 | S. W. | Clouds. Rainy night |
| 14 ☽ 47′ | 12 | 57 | 37 | 30 04 | 29 68 | S. W. | Clouds & rain, *a. m.* Fair, *p. m.* |
| | 13 | 43 | 37 | 30 02 | 30 02 | N. E. | Cloudy |
| | 14 | 41 | 31½ | 30 03 | 29 89 | N. E. | Fair day |
| | 15 | 36 | 32 | 29 89 | 29 58 | S. E. | Cloudy |
| | 16 | 39 | 32½ | 29 67 | 29 58 | N. E.—E. | Fair morning. Cloudy night |
| | 17 | 41 | 27 | 29 93 | 29 70 | N. E. | Fair |
| | 18 | 40 | 24 | 30 02 | 29 93 | N.E.–S.E. | Fair |
| | 19 | 48 | 31 | 30 10 | 30 07 | W.—N. | Fair |
| 14 ○ 31′ | 20 | 52 | 31 | 29 98 | 29 78 | N. W. | Fair |
| | 21 | 50 | 35 | 29 97 | 29 74 | W. N. W. | Fair |
| | 22 | 42 | 24 | 29 98 | 29 97 | N. | Fair |
| | 23 | 49 | 25 | 29 98 | 29 80 | S.—N. | Fair |
| | 24 | 46 | 35 | 29 83 | 29 80 | E. | Fair, and strong wind |
| | 25 | 40 | 31 | 29 96 | 29 84 | E. | Fine windy day |
| | 26 | 40 | 34 | 29 95 | 29 80 | E. S. E. | Clear windy day |

**THERMOMETER.**

Highest, March 10 — 58°
Lowest, March 22 — 24°
Mean of Observations for this period - 42°

**BAROMETER.**

Highest, Feb. 28 — 30.07
Lowest, March 7 — 28.85
Mean of Observations for this period - 29.69

### OBSERVATIONS.

Feb. 26. *Cirro-Cumuli* observed in the morning.

28. *Cirro-Stratus* observed in the west.

Mar. 3. Remarkably fine golden coloured sun-set.

5. Four different modifications of clouds seen at once, namely *Cirrus, Cirro-Cumulus, Cirro-Stratus,* and *Cumulus*; succeeded by rain during the night.

10. The Bat, *Vespertilio Murina*, first seen this year.

14. A lunar halo, about 40 deg. in diameter, seen from 8 till 12 o'clock.

24. The modification of *Cirrus* observable; these clouds, called by the country people *Mare's Tails*, usually precede high wind.

25. High wind all day, *Cirrus* still observable.

